# Phytochemical and Analytical Characterization of Novel Sulfated Coumarins in the Marine Green Macroalga *Dasycladus vermicularis* (Scopoli) Krasser

**DOI:** 10.3390/molecules23112735

**Published:** 2018-10-23

**Authors:** Anja Hartmann, Markus Ganzera, Ulf Karsten, Alexsander Skhirtladze, Hermann Stuppner

**Affiliations:** 1 Institute of Pharmacy, Pharmacognosy, CMBI, University of Innsbruck, Innrain 80-82, 6020 Innsbruck, Austria; markus.ganzera@uibk.ac.at (M.G.); hermann.stuppner@uibk.ac.at (H.S.); 2 Institute of Biological Sciences, Applied Ecology & Phycology, University of Rostock, Albert-Einstein-Str. 3, 18059 Rostock, Germany; ulf.karsten@uni-rostock.de; 3 Department of Phytochemistry, Iovel Kutateladze Institute of Pharmacochemistry, Tbilisi State Medical University, 0159 Tbilisi, Georgia; aleksandre.skhirtladze@yahoo.com

**Keywords:** siphonous green algae, sulfated coumarins, *Dasycladus vermicularis*, isolation and quantification

## Abstract

The siphonous green algae form a morphologically diverse group of marine macroalgae which include two sister orders (Bryopsidales and Dasycladales) which share a unique feature among other green algae as they are able to form large, differentiated thalli comprising of a single, giant tubular cell. Upon cell damage a cascade of protective mechanisms have evolved including the extrusion of sulfated metabolites which are involved in the formation of a rapid wound plug. In this study, we investigated the composition of sulfated metabolites in *Dasycladus vermicularis* (Dasycladales) which resulted in the isolation of two phenolic acids and four coumarins including two novel structures elucidated by nuclear magnetic resonance spectroscopy (NMR) as 5,8′-di-(6(6′),7(7′)-tetrahydroxy-3-sulfoxy-3′-sulfoxycoumarin), a novel coumarin called dasycladin A and 7-hydroxycoumarin-3,6-disulfate, which was named dasycladin B. In addition, an analytical assay for the chromatographic quantification of those compounds was developed and performed on a reversed phase C-18 column. Method validation confirmed that the new assay shows good linearity (R^2^ ≥ 0.9986), precision (intra-day R.S.D ≤ 3.71%, inter-day R.S.D ≤ 7.49%), and accuracy (recovery rates ranged from 104.06 to 97.45%). The analysis of several samples of *Dasycladus vermicularis* from different collection sites, water depths and seasons revealed differences in the coumarin contents, ranging between 0.26 to 1.61%.

## 1. Introduction

*Dasycladus vermicularis* (Scopoli) Krasser is an evolutionarily ancient, small siphonous green alga, which is widely distributed throughout tropical to temperate regions such as several Atlantic islands (Canary islands, Madeira, Antilles), many regions in the Mediterranean Sea, Central America (Belize), Caribbean islands, South America (Brazil), and Asia (Japan, South China Sea, Philippines) [1]. This chlorophyte inhabits well-illuminated shallow waters (0.3–20 m) with high light exposures in the upper littoral zone on rocky substrates and are often covered by a thin layer of sediment [2]. To thrive under enhanced doses of UV radiation, photo-protective mechanisms are needed. In 1983, Menzel et al. [3] isolated and identified 3,6,7-trihydroxycoumarin (thyc) as a UV absorbing compound from *D. vermicularis* for the first time, which was also the first report of coumarins in algae. Subsequently, several studies have been carried out to investigate the relevance of thyc in *D. vermicularis.* They indicated an elevated excretion of thyc due to increasing UV exposure and temperatures suggesting that this compound is a natural sunscreen/UV protectant [4,5,6]. Another very remarkable characteristic of *D. vermicularis* is its morphology. *D. vermicularis* is a member of the so-called siphonous green macro algae comprising unique giant single cells without cross walls. These thalli can grow up to 10 cm long, gaining stability by surrounding themselves with a calcareous coating which supports the long unicellular algae with sufficient stability to grow upright. Siphonous algae typically contain a huge central vacuole and a thin layer of cytoplasm, the latter inhabiting multiple nuclei (Bryopsidales) or just one nucleus (Dasycladales) [7]. After cell damage, for example due to herbivory, a rapid wound closure is essential for the survival of such organisms. Therefore, immediately upon injury a cascade of biochemical reactions is induced to assimilate cellular contents into an insoluble wound plug initially formed by gelling, followed by a slower hardening process (1–2 h) [8,9]. This mechanism is indispensable to avoid cytoplasmic loss and limits the intrusion of extracellular components, herbivore attack or pathogenic invasion, which could otherwise result in high mortality rates [8,10]. A few years ago Welling et al. [8] investigated both the intact and wounded alga to monitor changes in chemical composition, which may be involved in the wound plug formation. This study surprisingly revealed the dominant secondary metabolite to be 6,7-dihydroxycoumarin-3-sulfate (dhycs) in the methanolic extracts of intact *D. vermicularis*, while the previously reported major compound 3,6,7-trihydroxycoumarin was only found in the methanolic extracts after wounding. Thus, the dhycs is supposed to act as a precursor and is transformed into the more active thyc in the presence of sulfatases. According to Welling et al. [9] thyc acts as an intermediate which is rapidly oxidized and serves as a protein cross-linker for the formation of a wound sealing co-polymer in combination with amino acid side chains from the alga. Such polymerization processes are known from other marine organisms, which are usually involved in bioadhesive processes that are needed for sessile marine organisms such as tubeworms and mussels to attach themselves to surfaces. For example, the common blue mussel *Mytilus edulis* uses a metal centered chelate that initiates the biopolymerisation process which includes secondary metabolites such as protein-bound-dopamine [11]. Sulfated secondary metabolites are widely distributed among marine species and are stored therein in a dormant state. They are then transformed enzymatically into more active metabolites, for example, psammaplin A sulfate from the sponge *Aplysinella rhax* or zosteric acid, an antifouling metabolite in the seagrass *Zostera marina*. Both metabolites are converted upon tissue disruption to their desulfated form thereby increasing activity as a defensive metabolite [12,13]. Kurth et al. [14] just recently revealed the presence of two sulfated phenolic acids in *D. vermicularis* namely 4-(sulfooxy)benzoic acid (SBA) and 4-(sulfooxy)phenylacetic acid (SPA), which are also proposed to exist in a dormant state prior to transformation to more active desulfated metabolites. However, these two sulfated phenolic acids are most likely not involved in the wound plug formation, but could be serving as biofilm inhibitor. In this study we investigated the phytochemical composition of *D. vermicularis* extracts of different polarities. The coumarin composition of this alga seems to be more complex than previously described, and hence we report on two novel coumarins from *D. vermicularis* and their analysis via HPLC-MS and NMR. Coumarins are natural benzopyrone derivatives with a variety of desirable pharmacological properties which are commonly found in higher plants [15], however sulfated molecules are rather uncommon and beyond that *D. vermicularis* is the first alga known to contain these secondary metabolites. Therefore, we report on the first validated HPLC assay for the separation and quantification of sulfated coumarins in algae which has been applied to samples from three different sampling sites across the Mediterranean Sea to compare their coumarin content.

## 2. Results

### 2.1. Isolation and Identification of the Coumarins

With the aim of isolating the major compounds present in *Dasycladus vermicularis* four coumarins and two phenolic acids were isolated from the methanol and the aqueous extract. A sample (2.5 g) of the aqueous extract was separated by means of repeated flash chromatography and semi-preparative HPLC, which resulted in the isolation of compounds **1**, **4** and **8**. Compounds **2**, **3** and **7** were obtained from 4.5 g of crude methanol extract by silica gel and Sephadex LH 20 column chromatography. ^1^H- and ^13^C-NMR shift values of all isolated coumarins are summarized in Table 1. The shift values for thyc were in good agreement with literature values [16]. Compounds **1** and **2** were identified as new natural products.

Compound **1** was isolated as a yellowish amorphous powder. Its molecular formula was established as C_18_H_10_O_16_S_2_ by HR-ESI–MS (*m*/*z* 544.934, calcd. for [C_18_H_10_O_16_S_2_-H]^−^, 544.936) indicating the presence of a sulfated dicoumarin. Fragments at *m*/*z* 464.9 [M-H-80]^−^ and 385.0 [M-H-80-80]^−^ could be attributed to the loss of sulfate groups, respectively. The melting point was measured to as 278–280 °C The IR spectra shows a strong and characteristic S=O stretching vibration at about 1038 cm^−1^ for the R-OSO_3_H groups, 1689 and 1629 cm^−1^ for (C=O), 3059 and 3203 cm^−1^ (OH). In the ^1^H-NMR (Table 1) spectrum, four singlet aromatic proton resonances at δ*_H_* 7.96 (1H, s, H-4′), 7.40 (1H, s, H-4), 7.28 (1H, s, H-5′), 7.10 (1H, s, H-8) were observed. The ^13^C-NMR (Table 1) spectrum showed eighteen carbon signals, which were assigned by DEPT experiments to four aromatic methines and fourteen quaternary carbons, two of which (δ_C_ 163. 3 and 163.0) could be attributed to intramolecular ester groups. The spectroscopic data suggested that **1** is composed of two identical coumarin moieties substituted in positions C-3(C-3′), C-6(C-6′), and C-7 (C-7′). The absence of corresponding signals in the ^1^H-NMR spectrum and the low field-shifted C-5 and C-8′ signals in the ^13^C-NMR spectrum indicated that the two symmetric coumarin moieties were connected at 5–8′ through a C-C bond. Localization of the two sulfate groups was established based on the low field-shifted signals of C-3 and C-3′ as well as by comparison of NMR data with those of thyc (**4**) [16] and compound **3**. HSQC and HMBC experiments of **1** were useful to assign all signals in the ^1^H and ^13^C spectra. C-4 (δ 134.4) and C-8 (δ 106.8) were used to assign the H-4 (7.40, s) and H-8 (7.10, s) as well as C-4′ (δ 135.8) and C-5′ (δ 115.8) which were assigned to H-4′ (7.96, s) and H-5′ (7.28, s) by ^1^H-^13^C HSQC.

The HMBC spectrum showed key correlation peaks between the proton signal at δ*_H_* 7.93 (1H, s, H-4′) and carbon resonances at δ*_C_* 163.0 (C-2′), 147.7 (C-9′), 135.8 (C-3′), 115.8 (C-5′); and between the proton signal at δ*_H_* 7.38 (1H, s, H-4) and carbon resonances at δ*_C_* 163.3 (C-2), 150.3 (C-9), 136.1 (C-3), 118.3 (C-5). Thus, compound **1** was identified as 5,8′-di-(6(6′),7(7′)-tetrahydroxy-3-sulfoxy-3′-sulfoxycoumarin), a new coumarin for which we propose the trivial name “dasycladin A”.

Compound **2** was also isolated as a yellowish amorphous powder. The molecular formula of C_9_H_6_O_11_S_2_ was determined by HR-ESI–MS (negative mode) with a mass peak at *m*/*z* 352.928 (calcd. for [C_9_H_6_O_11_S_2_-H]^−^, 352.926). The mass spectrum showed a fragment at *m*/*z* 272.8 [M-H-80]^−^ corresponding to the loss of a sulfate moiety. The melting point was measured as 233–238 °C. The IR spectra shows a strong and characteristic S=O stretching vibration at about 1038 cm^−1^ for the R-OSO_3_H groups, 1697 cm^−1^ for (C=O), 3217 cm^−1^ (OH). The ^1^H-NMR spectrum (Table 1) of **2** showed signals for three aromatic protons at δ_H_ 7.94 (1H, s, H-4), 7.64 (1H, s, H-5), 7.05 (1H, s, H-8). In the ^13^C-NMR (Table 1) spectrum nine carbon signals, including three aromatic methines and six quaternary carbons were observed. HSQC and HMBC experiments of Compound **2** were useful to assign all signals in the ^1^H- and ^13^C-NMR spectra. C-4 (δ 133.3), C-5 (δ 122.4) and C-8 (δ 105.2) were used to assign the protons H-4 (7.94, s), H-5 (7.64, s) and H-8 (7.05, s) by ^1^H-^13^C HSQC. The HMBC spectrum showed correlation peaks between the proton signal at δ*_H_* 7.94 (1H, s, H-4) and carbon resonances at δ*_C_* 161.1 (C-2), 151.1 (C-9), 134.2 (C-3), 122.4 (C-5); between the proton signal at δ*_H_* 7.64 (1H, s, H-5) and carbon resonances at δ*_C_* 152.9 (C-7), 151.1 (C-9), 137.7 (C-6), 133.3 (C-4); and between the proton signal at δ*_H_* 7.04 (1H, s, H-8) and carbon resonances at δ*_C_* 152.9 (C-7), 151.1 (C-9), 137.7 (C-6), 112.3 (C-10). These spectroscopic data suggested that compound **2** is 3,6,7-trisubstituted coumarin. The low field-shifted signal of C-3 and the up field-shifted of C-6 indicated that the sulfate groups were attached at these carbons. The structure of **2** was deduced as 7-hydroxycoumarin-3,6-disulfate. Compound **2** represents a new coumarin for which we propose the trivial name “dasycladin B”.

NMR spectra and HR-ESI–MS data for the novel sulfated coumarins **1** and **2** are shown in Figures S1A–E and S2A–E in the Supplementary Material.

### 2.2. HPLC-Method Development

**Figure 1:**
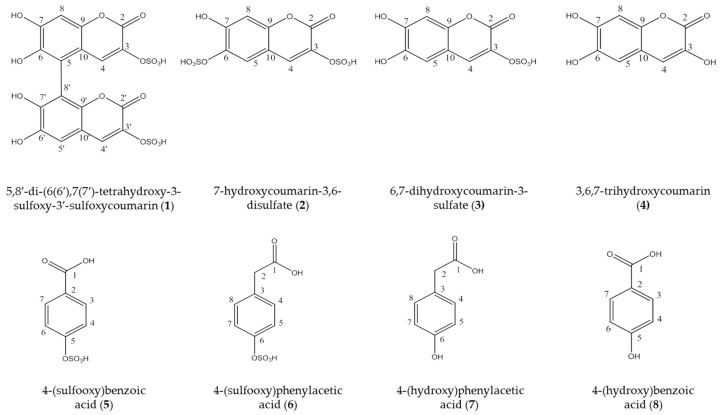
Chemical structure of the isolated coumarins from the marine green alga *Dasycladus vermicularis*, the sulfated phenolic acids and their educts.

**Figure 2:**
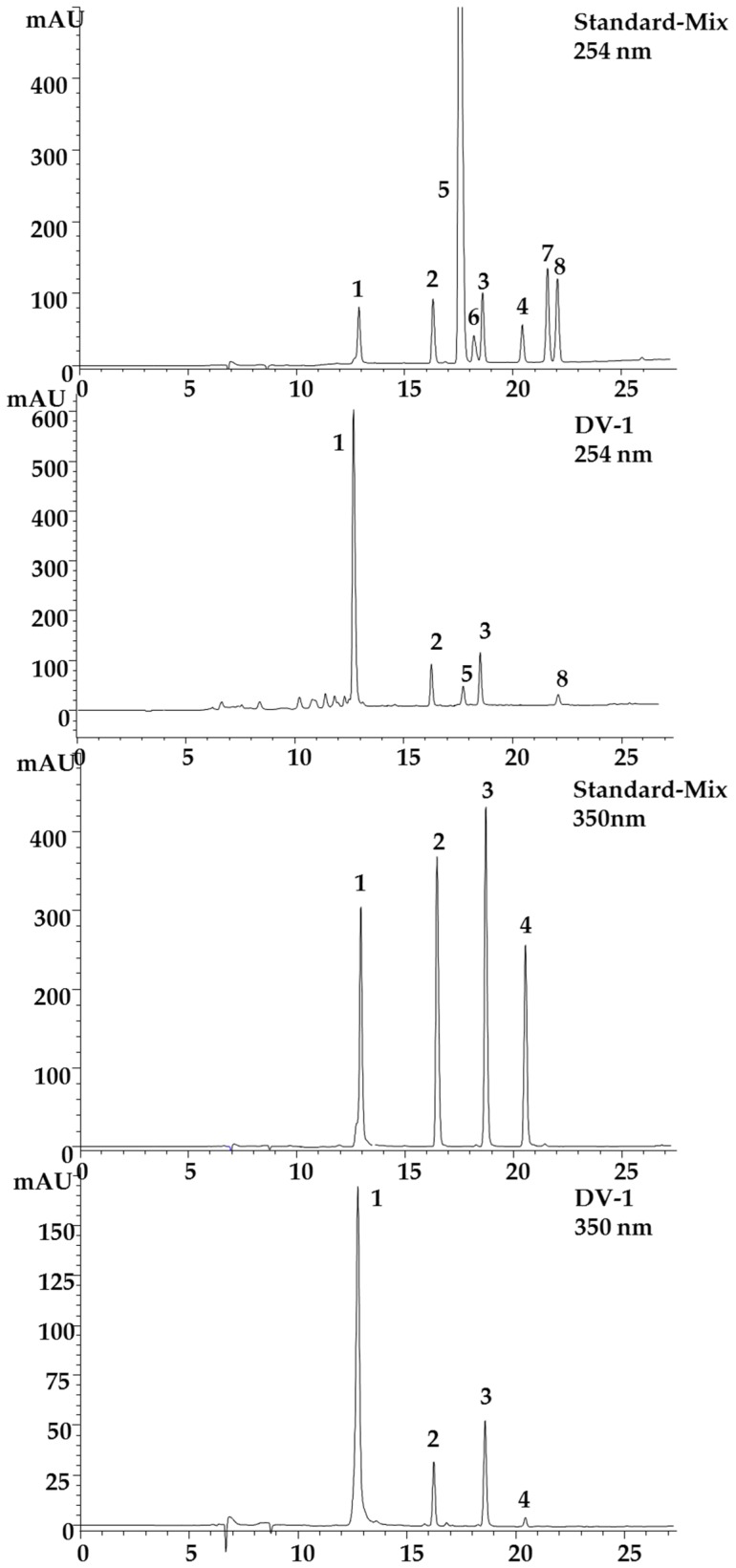
HPLC separation of all standards **1** to **8** at 254 nm and all coumarins **1** to **4** isolated from the marine green alga *Dasycladus vermicularis* at 350 nm, and the sample DV-1 from the marine green alga *Dasycladus vermicularis* at 254 nm and 350 nm under optimized conditions (column: Gemini C18, 110 Å column (150 × 4.6 mm, 3 μm); mobile phase (A) aqueous 20 mM ammonium acetate solution with 1.5% acetic acid and (B) methanol/water (9:1) with 20 mM ammonium acetate and 1.5% acetic acid. Gradient: 2% B to 15% B from 0 to 5 min and 15% B to 60% B from 5–20 min and 60% B to 98% B from 20–25 min; detection at 254 nm and 350 nm, flow rate 0.3 mL/min, injection volume 5 μL and 40 °C oven temperature. Peak assignment is according to Figure 1.

A HPLC method was developed for quantification of the coumarins. Four coumarins were isolated as described above and used as standards in addition to the two synthetized sulfated phenolic acids and their educts (see Figure 1). Several different stationary phases were screened for the separation of the coumarins and phenolic acids in *D. vermicularis*, such as Zorbax SB-C18 3.5 μm, Hyperclone ODS 3 μm, YMC-triart C-18, 3.5 μm and Kinetex C-18 2.6 μm. However, the best separation was achieved on the Gemini C 18 110 Å, 3 μm (150 mm × 4.6 mm). The latter column yielded the best results concerning separation efficiency and peak shape, resulting in an optimum separation within less than 25 min (Figure 2).

5,8′-Di-(6(6′),7(7′)-tetrahydroxy-3-sulfoxy-3′-sulfoxycoumarin) (**1**) eluted first (13.12 min), followed by 7-hydroxycoumarin-3,6-disulfate (**2**; 16.58 min), the sulfated phenolic acids 4-(sulfooxy)benzoic acid and 4-(sulfooxy)phenylacetic acid (**5**; 17.73 min, **6**; 18.3 min), then 6,7-dihydroxycoumarin-3-sulfate (**3**; 18.81 min), 3,6,7-trihydroxycoumarin (**4**; 20.63 min), 4-(hydroxyl)phenylacetic acid (**7**; 21.62 min), and finally 4-(hydroxyl)-benzoic acid (**8**; 22.07 min).

### 2.3. Method Validation

The new analytical method was validated according to the ICH guidelines [17] by establishing calibration curves of the two coumarin standards **1** and **4**, as well as the phenolic acids 4-hydroxy-benzoic acid and 4-hydroxyphenylacetic acid and their sulfated products 4-(sulfooxy)benzoic acid (SBA) and 4-(sulfooxy)phenylacetic acid (SPA) **5**–**8**. Sufficient material was not available for **2** and **3**. Excellent determination coefficients (R^2^ ≥ 0.9986) were obtained within a concentration range of 0.859–1154 μg/mL. Individual calibration levels were obtained by serial dilution, and each solution was analyzed under optimum HPLC conditions in triplicate. Limit of detection (LOD) and limit of quantification (LOQ) were calculated using defined concentration equivalents to S/N ratios of 3 (LOD) and 10 (LOQ). LOD and LOQ values ranged from 0.014–1.939 μg/mL, and from 0.044–5.876 μg/mL, respectively (Table 2). Selectivity of the method was assured by no visible co-elution (shoulders) in the relevant signals, LC-MS data, and by very consistent UV-spectra (as confirmed by the peak purity option in the operating software). The methods precision was confirmed by its repeatability, as well as inter- and intra-day variation which were determined in *D. vermicularis* sample (DV-2). For this purpose five individual samples at 250 mg/25 mL were extracted and analyzed on each of three consecutive days (Table 3). Intra-day (RSD ≤ 5.99%) and inter-day precision (RSD ≤ 7.49%) were within accepted limits. Accuracy was assured by spiking accurately weighed samples of DV-2 with three different concentrations of the standard substances. For all compounds the observed recovery rates were acceptable and ranged from 95.6 to 104.6%. Only for 3,6,7-trihydroxcoumarin the value for the low spike was at 91.3% (Table 3). This compound appeared to be stable in solution for at least several h, however when added to the extract it degrades rapidly and therefore the recovery rates are poor, especially at the lower concentrations.

### 2.4. Analysis of Samples

**Figure 3:**
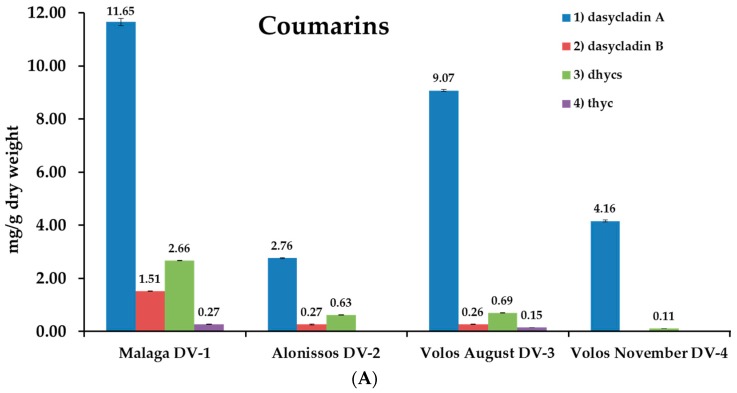

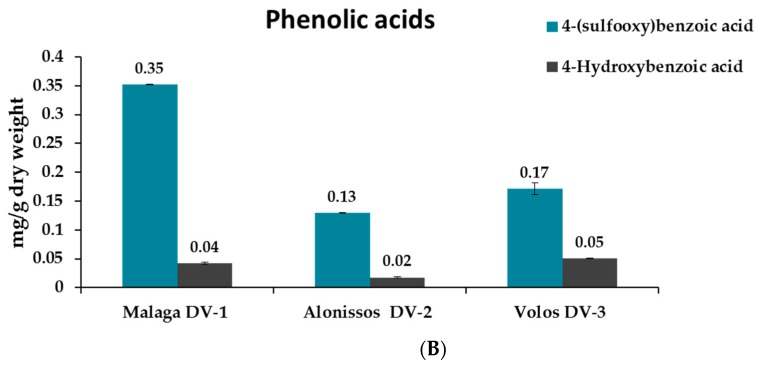
Quantification of the coumarins **1**) dasycladin A, **2**) dasycladin B, **3**) 6,7-dihydroxycoumarin-3-sulfate, **4**) 3,6,7-trihydroxycoumarin. Concentrations are given as mg coumarins/g dry weight (**A**) and as mg/g phenolic acids as dry weight (**B**) (n = 3) in the green alga *Dasycladus vermicularis* collected at different sampling sites and dates in the Mediterranean Sea.

**Figure 4:**
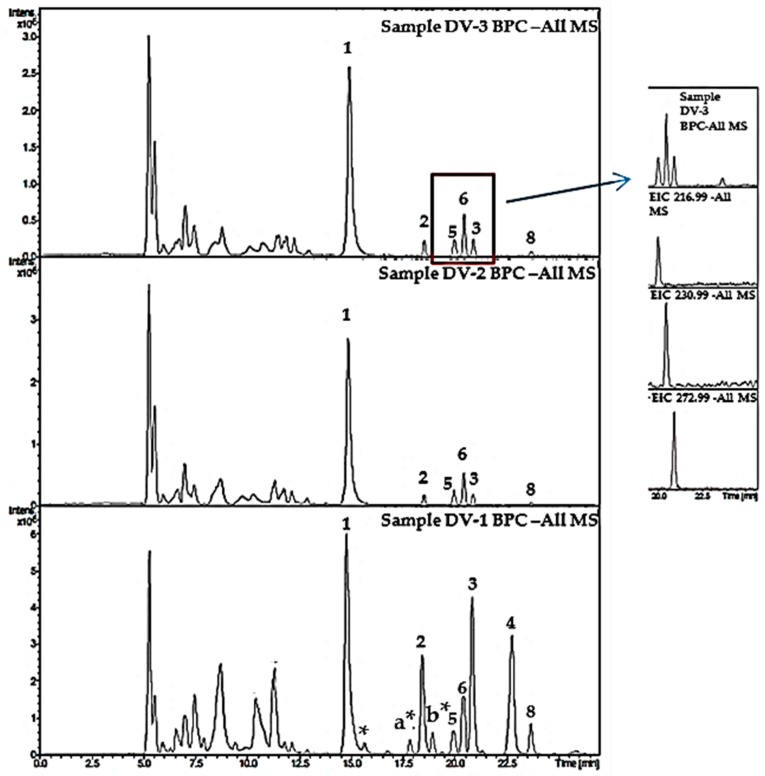
Base peak chromatograms of all extracts DV1-DV3 prepared from the marine green alga *Dasycladus vermicularis*. The red area is enlarged and shows the extracted ion chromatograms (EIC) for the sulfated phenolic acids SBA (**5**) and SPA (**6**). Tentatively identified minor coumarins are marked with a star (similar UV spectra) and with a* and b* (similar UV spectra and molecular weight).

Four different samples of *D. vermicularis*, all originating from the Mediterranean Sea, were analyzed for their coumarin content. For further details on collection sites, seasons and water depths see Supplementary Material Table S1. As expected, the total amount of coumarins in sample DV-2 (1.60–1.80 m depth) was much lower (3.66 mg/g DW) compared to sample DV-3 (0.30–0.80 m depth; 10.17 mg/g DW). The quantity of the monomeric sulfated coumarins **2** and **3** was the same, while the content of the dimeric compound **1** was about three times higher in the sample that was collected from shallow waters. The two samples that were collected from the same spot in Volos, Greece (DV-3 and DV-4) indicated that there might be seasonal changes in the coumarin content of *D. vermicularis* as well. In November (sample DV-4; 4.27 mg/g DW), the total coumarin content was only half the amount as in August (sample DV-3; 10.17 mg/g DW). The sample that was harvested from Malaga, Spain (DV-1) showed the highest amount of coumarins with 16.09 mg/g DW. The dimeric coumarin **1** was the most abundant compound in all samples, followed by **3**, the 6,7-dihydroxycoumarin-3-sulfate, and 7-hydroxycoumarin-3,6-disulfate (**2**). Thyc, which is reported to be the sole metabolite of compound **3**, only occurred in traces in the highest concentrated samples DV-1 and DV-3. Phenolic acids were also present in all samples, however, in much lower content. All samples contained 4-hydroxybenzoic acid and at a higher concentration its sulfated precursor 4-(sulfooxy)benzoic acid. All quantitative results are summarized in Figure 3. 4-(Sulfooxy)phenylacetic acid which was reported to be present in *D. vermicularis* in a previous publication by Kurth et al. [14] could not be detected in the samples by DAD; however, LC-MS revealed the presence of this compound in all samples. In addition to the standard compounds, three other signals were tentatively identified as coumarins based on the typical UV and MS-spectra; they are marked with a star (Figure 4). The compounds a* and b* both have UV absorption maxima of 346 nm and *m*/*z* of 465 [M-H^−^]. These two compounds may be monosulfated dicoumarins. The assignment of other signals was easily possible by comparison to standards. For example, the determination of coumarins in sample DV-1 is shown in Figure S5 in the Supplementary Material. Chromatograms were recorded at 254 nm and 350 nm, the other traces show the identification of individual compounds by LC-MS in EIC mode.

## 3. Discussion

The chemical composition of coumarins and phenolic acids in the marine green alga *D. vermicularis* was investigated in detail in this study, revealing that the coumarin composition is more complex than previously reported [8]. Dasycladin A is the major compound, which probably degrades to the previously reported 6,7 dihydroxycoumarin-3 sulfate. Similarly, it can be speculated that dasycladin B is metabolized to the corresponding monosulfated coumarin first and afterwards to the more active metabolite 3,6,7-trihydroxycoumarin, since the sulfated compounds have been reported as dormant forms which are enzymatically transferred into active metabolites through sulfatases [9]. The sulfated phenolic acids SPA and SBA were both present in the extract, however at very low concentrations. These findings are highlighted with red in Figure 4 and are in good agreement with the LC-MS data published by Kurth et al. [14]. Coumarins in general show a wide range of different pharmacological activities, including anti-HIV, anti-tumor, anti-hypertensive, anti-coagulant, anti-inflammatory just to name a few. They have become important lead compounds in drug research due to their high bioavailability, low molecular weight, and low toxicity [15,18]. For simple coumarins anti-oxidative activity has been reported especially for compounds with free hydroxyl groups. Likewise, the C-7 free hydroxyl group is important for anti-bacterial activity and is also important for an anti-inflammatory activity [19]. The C-6 free hydroxyl group is important for both anti-bacterial and anti-fungal activity. A free hydroxyl group in position 3 (e.g., Compound **4** in this study) is especially important for a strongly enhanced inhibition of 5-Lipoxygenase and α-D-glucosidase [20]. Sulfated coumarins are rather uncommon but due to their negative charge they could bind to heparin receptors or inhibit platelet aggregation [21].

Besides recently developed HPLC-DAD, LC-MS and SFC methods for the separation of furo- pyrano- and monocoumarins on reversed phase columns [22,23,24], this study presents the first method for the separation of the definitive more polar sulfated coumarins. Their content was found to be quite variable in the different samples suggesting that water depth and seasonal changes including fluctuations in the visible and ultraviolet part of solar radiation might have a strong influence. *D. vermicularis* has been reported to excrete UV-absorbing brown-green substances under in-situ conditions staining the nearby seawater and thus being beneficial as photo-protective compounds for other macroalgae living in the vicinity [6]. The responsible compounds were later identified as coumarins and since they are accumulated in the outer parts of the siphonous cell walls, particularly after UV exposure, they are considered to act as UV-sunscreens [6]. 3,6,7-trihydroxycoumarin (thyc) was found to be preferentially localized in the apical part of the *D. vermicularis* thallus, which usually experiences highest natural insolation, particularly in the internal part of the cell wall and around the tonoplast. The percentage of UVR absorbed by both thyc layers could be measured from the in-vitro total thallus concentration of thyc and histological measurements of these layers. While the cell wall thyc layer absorbed 88% of the incident UVR irradiance, the one close to the vacuole membrane absorbed a similar fraction with 87.5% [6]. These data strongly support the hypothesis of coumarins/phenolics as natural sunscreen compounds reducing biologically harmful UVR from reaching sensitive biomolecules in the cell such as DNA and proteins. Phenolic compounds also play an important role in the interaction of macroalgae with their environment. They are relevant for different supporting or protective tissues, for example in cell wall formation, they can be involved in defense mechanisms, for example in anti-herbivory or having antibacterial effects, and signaling properties, for example in allelopathy [25].

The conspicuous decrease of coumarin content in November could make *D. vermicularis* more susceptible to abiotic and biotic stressors, but UVR and biotic interactions are less strong in late autumn compared to summer in the Mediterranean Sea. However, a larger number of samples from different origins, seasons and water depths is definitely needed to examine the qualitative and quantitative metabolic composition of *D. vermicularis* and its chemical reaction after wounding or other stress situations.

## 4. Materials and Methods

### 4.1. Reagents and Chemicals

All solvents used for isolation, synthesis and analytical studies were of analytical grade and purchased from Merck (Darmstadt, Germany). HPLC grade water was produced by a Sartorius arium 611 UV water purification system (Sartorius Göttingen, Germany). Reagents for the synthesis of 4-(sulfooxy)benzoic acid and 4-(sulfooxy)phenylacetic acid (4-hydroxybenzoic acid, sulphur trioxide pyridine complex (Pyr*SO_3_), and 4-hydroxyphenylacetic acid) were purchased from Sigma Aldrich (Taufkirchen, Germany). Deuterated water for NMR experiments was obtained from Euriso-top (Saint-Aubin Cedex, France).

### 4.2. Algal Material

Four samples of *Dasycladus vermicularis* were analyzed. DV-1(330 g dry weight) was collected in September 1998 from the upper part of the infralittoral zone (0.5 m depth) in the Cabo de Gata-Nijar Natural Park (36° 52′ N, 2° 12′ W, Almerıa, Southern Spain) and identified by Prof. Felix Figueroa (University of Malaga), freeze-dried and sent to Ulf Karsten, who stored the material under cool, dry and dark conditions prior to further processing in Innsbruck. Sample DV-2 was harvested in August 2017 in Alonissos, Greece (39°08′23.8′′N, 23°50′43.3′′ E) at 1.80 m depth; Sample DV-3 and DV-4 were both harvested in Volos, Greece (39°1900.0′′N, 23°01′11.5′′ E), DV-3 in August 2017 and DV-4 in November 2017 at 30–80 cm depth. All samples from Greece were collected and identified by the author (A. Hartmann). All samples were air-dried and voucher specimen of all samples are stored at the Institute of Pharmacy, Pharmacognosy, University of Innsbruck.

### 4.3. Instrumentation

NMR experiments were conducted on an Avance II 600 spectrometer (Bruker, Karlsruhe, Germany) operating at 600.19 (^1^H) and 150.91 MHz (^13^C). Spectra of the respective compounds were recorded in deuterated solvents from Euriso-Top adding 3-(trimethylsilyl)-propionic acid sodium salt (TMSP) as an internal standard. Infrared (IR) spectra were recorded on an ALPHA Fourier transform (FT)-IR apparatus (Bruker, Billerica, MA, USA) equipped with a platinum attenuated total reflection module. Analytical HPLC experiments were carried out on an Agilent 1100 system (Agilent, Waldbronn, Germany) equipped with a binary pump, autosampler, diode array detector and column oven. For the purification of the compounds a semi-preparative HPLC from Dionex (ThermoFisher, Waltham, MA, USA), comprising of a HPG-3200 pump, a VWD-3100 detector, column oven and a fraction collector was utilized. Additionally, the exact mass of the novel compounds **1** and **2** were determined in negative ESI mode on a micrOTOF-Q II MS (Bruker, Bremen, Germany). The settings were: nebulizer gas: 4.4 psi, dry gas: 4 L/min, dry temperature: 180 °C, capillary voltage: 2.5 kV, set capillary V 3500, set endplate offset V-500.

### 4.4. Isolation and Structural Analysis of Coumarins

Dried algal material of *Dasycladus vermicularis* (300 g, DV-1, Malaga), were finely ground to powder and subsequently extracted five times with 100% dichloromethane (p.a.) for 15 min in an ultrasonic bath. After centrifugation at 1537× *g*, the combined solutions were evaporated to dryness at 40 °C under reduced pressure to yield 1.13 g of crude dichloromethane extract (DV-1D). The plant material was subsequently extracted with 100% methanol (p.a.) using the same procedure to yield 4.71 g of crude methanol extract (DV-1M). The algal residue was extracted for a third time using the same procedure with a 1:1 mixture of water and methanol to yield 7.9 g crude aqueous extract. The crude methanol extract (4.5 g) was separated into 10 fractions (DV-M-S1-10) on silica gel (40–63 μm particle size) using a dichloromethane/ethyl acetate/methanol/water gradient. Fraction DasyM-S7 was further purified using size-exclusion chromatography on Sephadex LH-20 material with methanol:water (1:1) as eluent to obtain 16 subfractions (DV-M 7.1-16). This resulted in the isolation of compounds **2** (5.24 mg) and **3** (4.04 mg). A portion (2.5 g) of the crude aqueous extract was first separated using flash chromatography (on RP-18 material (80 g Reveleris cartridge, 40 μm) and a water/methanol gradient, containing 0.25% formic acid in each solvent. 20 subfractions were obtained (DV-W-R1-20). Fraction 18 resulted in the pure compound **4**. DV-W-R6 (39.56 mg) was purified by semi-preparative HPLC on a Lichrosorb RP-18 (250 × 10 mm, 7 μm) column with a gradient of 2–50 % methanol in water within 25 min at a flow of 1 mL/min. The oven temperature was set to 20 °C and the UV-detector signal to 350 nm, resulting in 7.91 mg of compound **1**. Fraction DV-W-R14 (123 mg) was re-chromatographed on a smaller C18 column using flash chromatography which resulted in 2.6 mg of compound **8**. The samples were dissolved in deuterated water with sodium trimethylsilyl propionate (TSP) as internal standard and in case of 3,6,7-trihydroxycoumarin deuterated methanol was used. NMR data of the isolated coumarins are summarized in Table 1. The shift values for thyc were in good agreement to literature values [16]. NMR spectra for the novel sulfated coumarins **1** and **2** are shown in Figure 1A–D and Figure 2A–D in the Supplementary Material. Compounds **3** and **4** are known natural products. Their structures were identified by comparison of their reported spectroscopic data, including ESI-MS and NMR data: 

*6,7-Dihydroxycoumarin-3-sulfate* (**3**, Figure 1): UV_max_ 268, 346 nm. It was assigned with a molecular formula C_9_H_5_O_8_S of ESI-MS, *m*/*z* 272.98 [M − H]^−^. ^1^H-NMR (600 MHz, D_2_O + 0.05% TSP) *δ*(ppm): 7.87 (s, 1H), 7.10 (s, 1H), 6.95 (s, 1H) ^13^C NMR *δ*(ppm): 163.6 (C-2), 135.9 (C-3), 135.8 (C-4), 115.7 (C-5), 145.1 (C-6), 151.8 (C-7), 106.0 (C-8), 149.7 (C-9), 114.2 (C-10) [9].

*3,6,7-Trihydroxycoumarin* (**4**, Figure 1): UV_max_ 268, 346 nm. It was assigned with a molecular formula C_9_H_6_O_5_ of ESI-MS, *m*/*z* 193.14 [M − H]^−^. ^1^H-NMR (600 MHz, D_2_O + 0.05% TSP) *δ*(ppm): 6.94 (s, 1H), 6.79 (s, 1H), 6.73 (s, 1H) ^13^C-NMR *δ* (ppm): 161.7 (C-2), 140.3 (C-3), 117.6 (C-4), 111.6 (C-5), 144.5 (C-6), 148.0 (C-7), 103.5 (C-8), 145.3 (C-9), 113.9 (C-10) [16].

### 4.5. Sample Preparation

The powdered dried alga (200 mg) was extracted three times with 8 mL of water: methanol (1:1) each by 15 min of sonication (Sonorex 35 KHz, Bandelin, Berlin, Germany). After centrifugation (1000× *g* for 3 min), the supernatants were combined in a 25 mL volumetric flask. Samples were measured immediately after extraction. 

### 4.6. Analytical Conditions

Experiments were performed on an Agilent 1100 HPLC system using a Gemini C18, 110 Å column (150 × 4.6 mm, 3 μm) from Phenomenex (Aschaffenburg, Germany). The mobile phase (A) contained water with 20 mM ammonium acetate and 1.5% acetic acid and (B) methanol/water (9:1) with 20 mM ammonium acetate and 1.5% acetic acid. Elution was performed in gradient mode starting with 2% B to 15% B from 0 to 5 min, 15% B to 60% B from 5–20 min and 60% B to 98% B from 20–25 min, followed by 10 min of re-equilibration with 98% A. The DAD was set to 254 nm and 350 nm, and flow rate, sample volume and column temperature were adjusted to 0.3 L/min, 5 μL and 40 °C, respectively. HPLC-MS experiments were carried out on an Agilent 1260 HPLC system coupled to an amaZon iontrap mass spectrometer (Bruker, Bremen, Germany). The chromatographic conditions were as described before; MS-spectra were recorded in negative ESI mode, with a drying gas temperature of 220 °C, the nebulizer gas (nitrogen) set to 23 psi, and a nebulizer flow (nitrogen) of 6 L/min. The scanned mass range was between *m*/*z* 70–1500, at a capillary voltage of 4.5 kV.

### 4.7. Synthesis of 4-(sulfooxy)benzoic Acid and Synthesis of 4-(sulfooxy)phenylacetic Acid

The synthesis of the two sulfated phenolic acids **5** and **6** was carried out as described recently by Kurth et al. [14], however using smaller quantities. 4-Hydroxybenzoic acid (1.5 g), sulfur trioxide pyridine complex (Pyr^∗^SO_3_, 1.72 g) were dissolved in water free pyridine (25 mL) and stirred in a 250 mL round bottomed flask at 25 °C for 48 h. For the synthesis of SPA, 4-hydroxyphenylacetic acid (0.75 g) and sulfur trioxide pyridine complex (Pyr^∗^SO_3_, 0.83 g) were used. Subsequently, the pyridine was removed by evaporation at 40 °C under reduced pressure. The remaining yellow-brown oil was dissolved in water (20 mL, HPLC grade) and the solution adjusted to pH 6–7 (pH-Meter, Mettler Toledo, Greifensee, Switzerland) using 25% potassium hydroxide. The aqueous solution was washed three times in a separatory funnel. A white precipitate was formed and filtered off after phase separation. Subsequently, the clear aqueous solution was evaporated at 45 °C under reduced pressure to give a white residue, which was re-dissolved in water and adjusted to pH 10 again using 25% KOH. To cleave the anhydride side products this solution was stirred at 60 °C for 1 h and subsequently neutralized using diluted sulfuric acid and afterwards evaporated again. The yellow-white residue was suspended in 10 mL water at 40 °C (5 mL for the synthesis of SPA). A white precipitate was formed by adding 20 mL (10 mL) of methanol, which was filtered off and subsequently washed with 10 mL (5 mL) of methanol. The methanol fractions were combined and left at 4 °C for 24 h in the fridge, so that a crystalline precipitate was formed; it was filtered off and the solution evaporated to dryness. The so obtained raw product was suspended in the ultrasonic bath using 2 mL (1 mL) methanol (HPLC-grade), which resulted in a white powder in a yellow solution. This step was repeated twice. Finally the product was washed with acetone and dried at 70 °C to give 660.93 mg (44% yield) SBA and 253.86 mg SPA (33.8% yield).

SBA: ^1^H-NMR (600 MHz, D_2_O + 0.05% TSP) *δ*(ppm): 7.91 (d, 2H; *J* = 8.4 Hz); 7.36 (d, 2H; *J* = 8.4 Hz), ^13^C-NMR *δ*(ppm): 177.7(C, C-1), 123.9 (C, C-2), 136.8 (CH,C-3,C-7), 133.3 (CH, C-4,C-6), 156.4 (C, C-5) HPLC–MS *m*/*z* [M − H]^−^: 216.99

SPA: ^1^H-NMR (600 MHz, D_2_O + 0.05% TSP) δ(ppm): 3.55 (s, 2H; *J* = 8.4 Hz), 7.26 (d, 2H; *J* = 8.5 Hz), 7.32 (d, 2H), ^13^C-NMR δ(ppm): 183.5 (C, C-1), 46.5 (CH_2_, C-2), 124.2 (C, C-3), 133.1 (CH,C5; CH, C7), 138.1 (CH,C4; CH,C8), 152.3 (C, C-6), HPLC–MS *m*/*z* [M − H]^−^: 231.00.

### 4.8. Synthesis of 3,6,7-trihydroxycoumarin

The synthesis of thyc was performed as previously published by Cotelle et al. [26] Briefly, 2,4,5 trihydroxybenzaldehyde (2.31 g), acetylglycine (2.10 g) and sodium acetate (1.59 g) were weighted into a round bottomed flask (100 mL), acetic anhydride (7.5 g) was added and the mixture was heated under reflux for 4 h (130 °C). The solution was cooled to room temperature and ice water (10 mL) was added. The precipitate was filtered, washed with ethanol/water (50:50) and dried to give 3-acetamido-6,7-diacetoxycoumarin. A solution of this intermediate (1.59 g) in 3 M HCl (50 mL) plus acetic acid (2 mL) was refluxed for 1 h and subsequently cooled to room temperature. The obtained precipitate was washed again with water to give pure 3,6,7-trihydroxycoumarin (667.2 mg).

## Figures and Tables

**Table 1:** NMR shift values of compounds isolated from the marine green alga *Dasycladus vermicularis*; spectra were recorded on a 600 MHz NMR instrument in deuterated water.

Position	Dasycladin A (1) in D_2_O		HMBC C	Dasycladin B (2) in D_2_O		HMBC C
	δ*_H_*	δ*_C_, *Type		δ*_H_*	δ*_C_, *Type	
2		163.3, C			161.0, C	
3		136.1, C			134.1, C	
4	7.40 (s)	134.4, CH	2, 3, 5, 9	7.94 (s)	133.3, CH	2, 3, 5, 9
5		118.3, C		7.64 (s)	122.4, CH	4, 6, 7, 9
6		144.1, C			137.6, C	
7		152.7, C			153.0, C	
8	7.10 (s)	106.8, CH	6, 7, 9, 10	7.05 (s)	105.1, CH	6, 7, 9, 10
9		150.2, C			151.1, C	
10		113.5, C			112.1, C	
2′		163.0, C				
3′		135.8, C				
4′	7.96 (s)	135.8, CH	2′, 3′, 5′, 9′			
5′	7.28 (s)	115.8, CH	4′, 6′, 7′, 9′			
6′		145.7, C				
7′		151.5, C				
8′		111.2, C				
9′		147.7, C				
10′		113.9, C				

**Table 2:** Calibration data for the coumarins isolated from the marine green alga *Dasycladus vermicularis* and the respective phenolic acids.

Parameter	1 (Dasycladin A)	4 (thyc)	5 (SBA)	6 (SPA)	7 (4-OH-PAA)	8 (4-OH-BA)
Regr. Equation	Y = 22.953x − 7.904	Y = 67.354x − 296.52	Y = 19.684x + 22.158	Y = 0.652x − 1.475	Y = 97.057x + 14.771	Y = 2.110x + 2.757
σ rel of the slope	0.09	0.683	0.116	0.175	0.049	0.339
R	0.9999	0.9986	0.9999	1.000	1.000	1.000
Range (μg/mL)	440–0.859	445–6.953	629–1.229	1154–18.031	124.75–0.975	483–3.770
LOD ^1^	0.192	0.589	0.039	1.939	0.014	1.045
LOQ ^2^	0.581	1.784	0.117	5.876	0.044	3.168

^1 ^LOD: limit of detection determined with purified standards (in μg/mL). ^2 ^LOQ: limit of quantification determined with purified standards (in μg/mL).

**Table 3:** Accuracy and precision of the new coumarin assay.

Substance	Accuracy ^1^			Precision ^2^			
	High Spike	Medium Spike	Low Spike	Day 1	Day 2	Day 3	Intra-Day
**1**	99.49	95.63	101.89	7.49	1.57	1.38	1.86
**2**	-	-	-	2.22	4.43	6.65	3.71
**3**	-	-	-	6.39	4.37	2.81	5.99
4	97.49	97.87	91.34	-	-	-	-
**5**	102.62	99.58	99.40	-	-	-	-
**6**	98.70	103.23	100.72	-	-	-	-
**7**	97.45	104.06	103.77	-	-	-	-
**8**	102.10	99.50	98.85	-	-	-	-

^1^ Expressed as recovery rates in percent (sample DV-2 Alonissos). ^2^ maximum relative standard deviation (peak area) within one and three consecutive days (n = 5; sample: DV-2).

## Supplementary Materials

The following are available online. Table S1: Origin of analyzed samples, Figure S2A–E: NMR spectra and HR-ESI-MS spectra of Compound **1**, Figure S3A–E: NMR spectra and HR-ESI-MS spectra of Compound **2,** Figure S4: UV spectra of the 4 Coumarins recorded on line by DAD, Figure S5: Determination of coumarins (**1**: dasycladin A, **2**: dasycladin B, **3**: 6,7-dihydroxycoumarin-3sulfate, **4**: 3,6,7-trihydroxycoumarin) in the marine green alga *Dasycladus vermicularis*.
