# Supplementary material for: Phytochemical and Analytical Characterization of Novel Sulfated Coumarins in the Marine Green Macroalga Dasycladus vermicularis (Scopoli) Krasser
Source: Molecules. 2018 Oct 23;23(11):2735. doi: 10.3390/molecules23112735 (PMC6278426; doi:10.3390/molecules23112735)
Supplement: Supplementary file 1 [file molecules-23-02735-s001.pdf]

Supplementary Information:

Phytochemical and analytical characterization of novel sulfated Coumarins in the marine green macroalga *Dasycladus vermicularis* (Scopoli) Krasser

Anja Hartmann [1*], Markus Ganzera[1], Ulf Karsten[2], Alexsander Skhirtladze [3] and Hermann Stuppner[1]

[1]Institute of Pharmacy, Pharmacognosy, CMBI, University of Innsbruck, Innrain 80-82, 6020 Innsbruck, Austria;
[2]Institute of Biological Sciences, Applied Ecology & Phycology, University of Rostock, Albert-Einstein-Str. 3, 18059 Rostock, Germany;
[3]Department of Phytochemistry, Iovel Kutateladze Institute of Pharmacochemistry, Tbilisi State Medical University, Tbilisi, Georgia.
*Correspondence: Anja.Hartmann@uibk.ac.at; Tel.: +43 512 507 – 58430

Received: date; Accepted: date; Published: date

Table S1: Origin of analyzed samples

Sample	Origin of sample
DV-1	September 1998, Cabo de Gata-Nijar Natural Park 36° 52′ N, 2° 12′ W, Almerıa, Southern Spain; 0.5 m depth
DV-2	August 2017, Great Mourtias 39°08'23.8"N, 23°50'43.3"E, Alonissos, Greece ; 1.60-1.80 m depth
DV-3	August 2017, Agios Minas 39°19'00.0"N, 23°01'11.5"E, Volos, Greece 0.3-0.8 cm depth
DV-4	November 2017, Agios Minas 39°19'00.0"N, 23°01'11.5"E, Volos, Greece 0.3-0.8 cm depth

According to CBD-Convention of Biological diversity and the ABSCH- the Access and Benefit-Sharing Clearing House [27, 28] Greece is not a party to the Nagoya protocol (ABSCH-MSR-GR-208044, Current status of the measure is not legally binding), however a MTA has been concluded between the University of Athens, Department of Pharmacy, Faculty of Pharmacognosy and Natural Product Chemistry and the Department of Pharmacognosy, University of Innsbruck. Samples were collected during a short term fellowship to the University of Athens funded by EMBO (ASTF 448 – 2016)

Spain is a party to the Nagoya protocol (ABSCH-MSR-ES-208924, current status legally binding since 15.05.2017). Samples from Spain were collected in 1998 before NP Nagoya protocol came into force by Professor Figueroa and have already been analyzed in previously published articles by him and Co-author Ulf Karsten [4, 29].

Figure S2: Figure A-D) NMR spectra and 1 E) HR-ESI–MS spectra for compound **1**:

15,8′-di-(6(6′),7(7′)-tetrahydroxy-3-sulfoxy-3′-sulfoxycoumarin).

A)

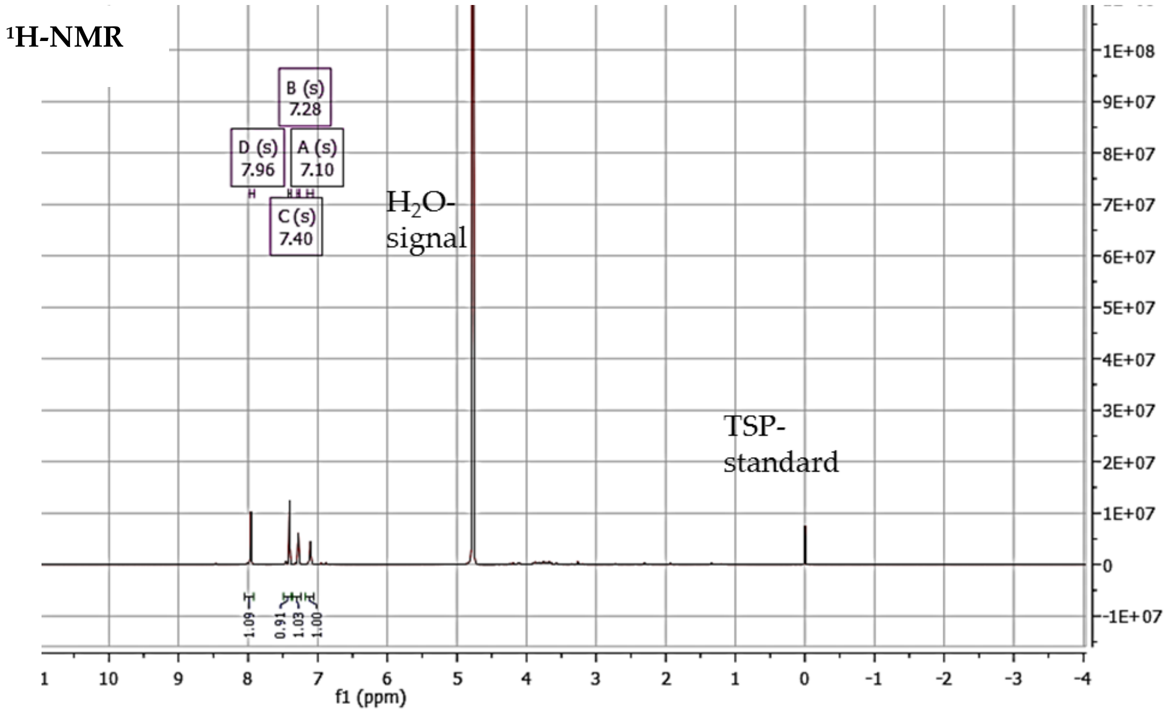

1H-NMR
B (s) 7.28
D (s) 7.96
A (s) 7.10
C (s) 7.40
H2O-signal
TSP-standard
1.09
0.91
1.03
1.00
f1 (ppm)

B)

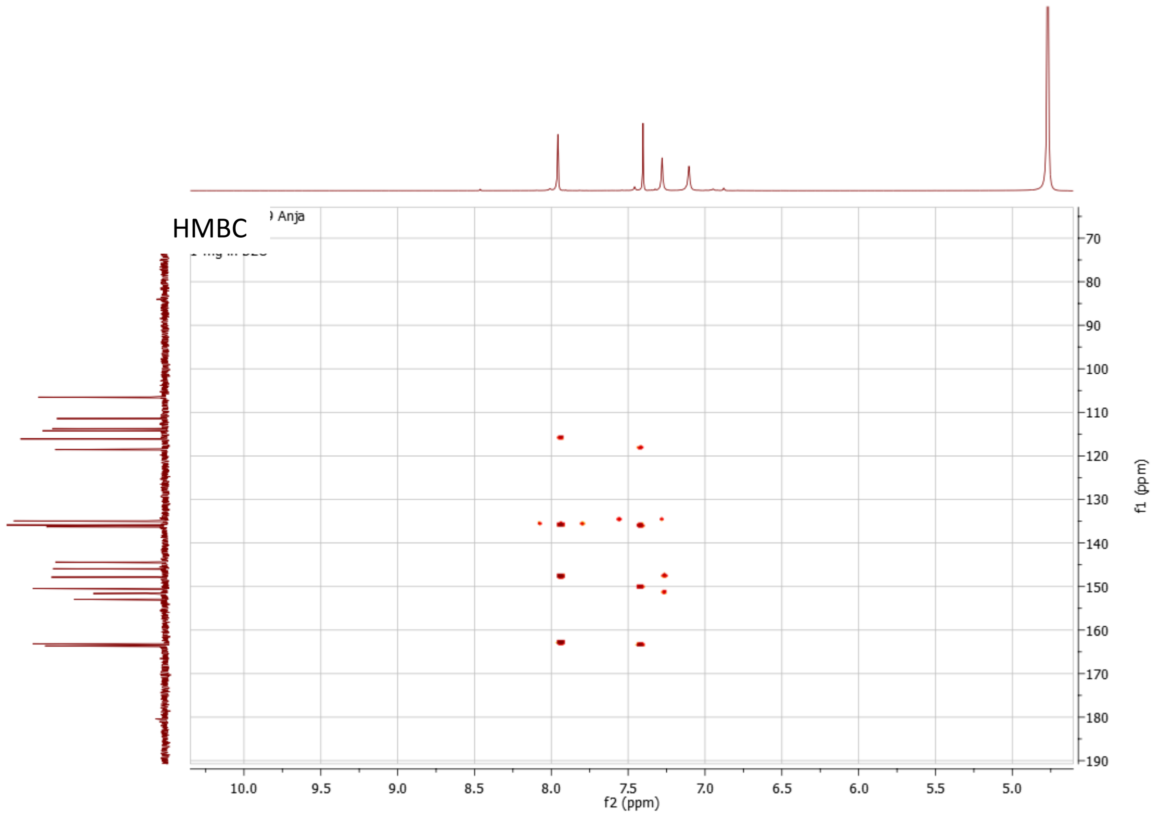

HMBC
f2 (ppm)
f1 (ppm)

C)

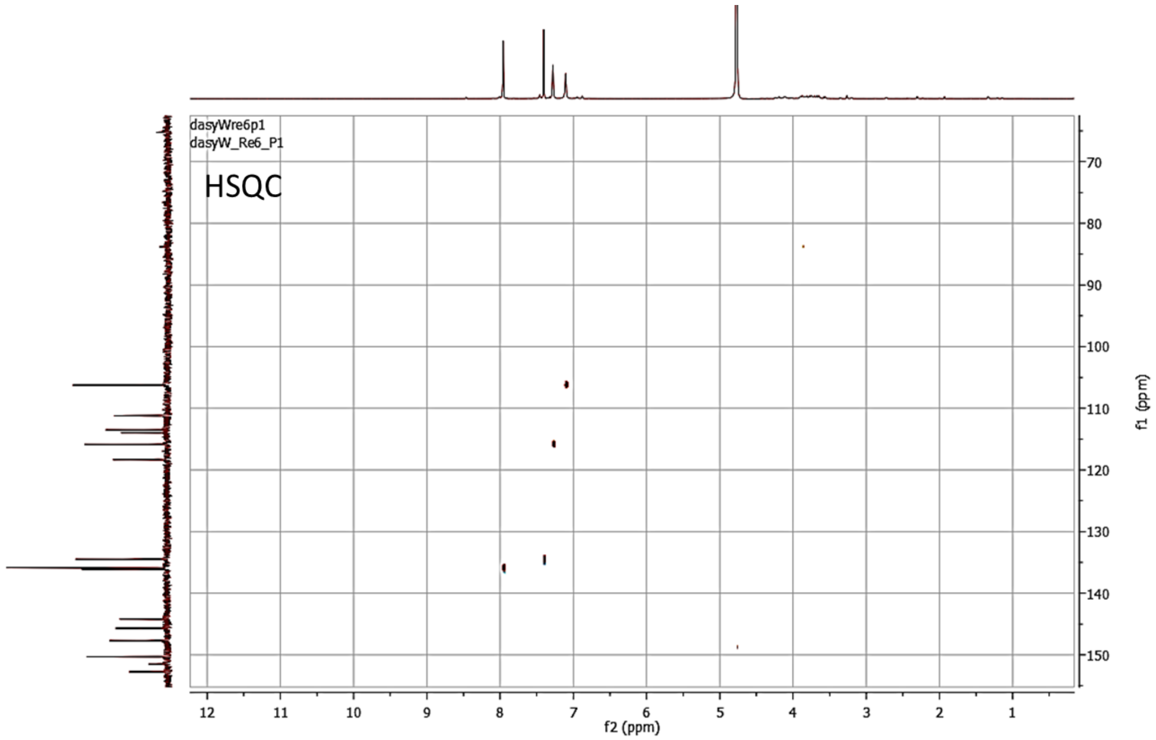

D)

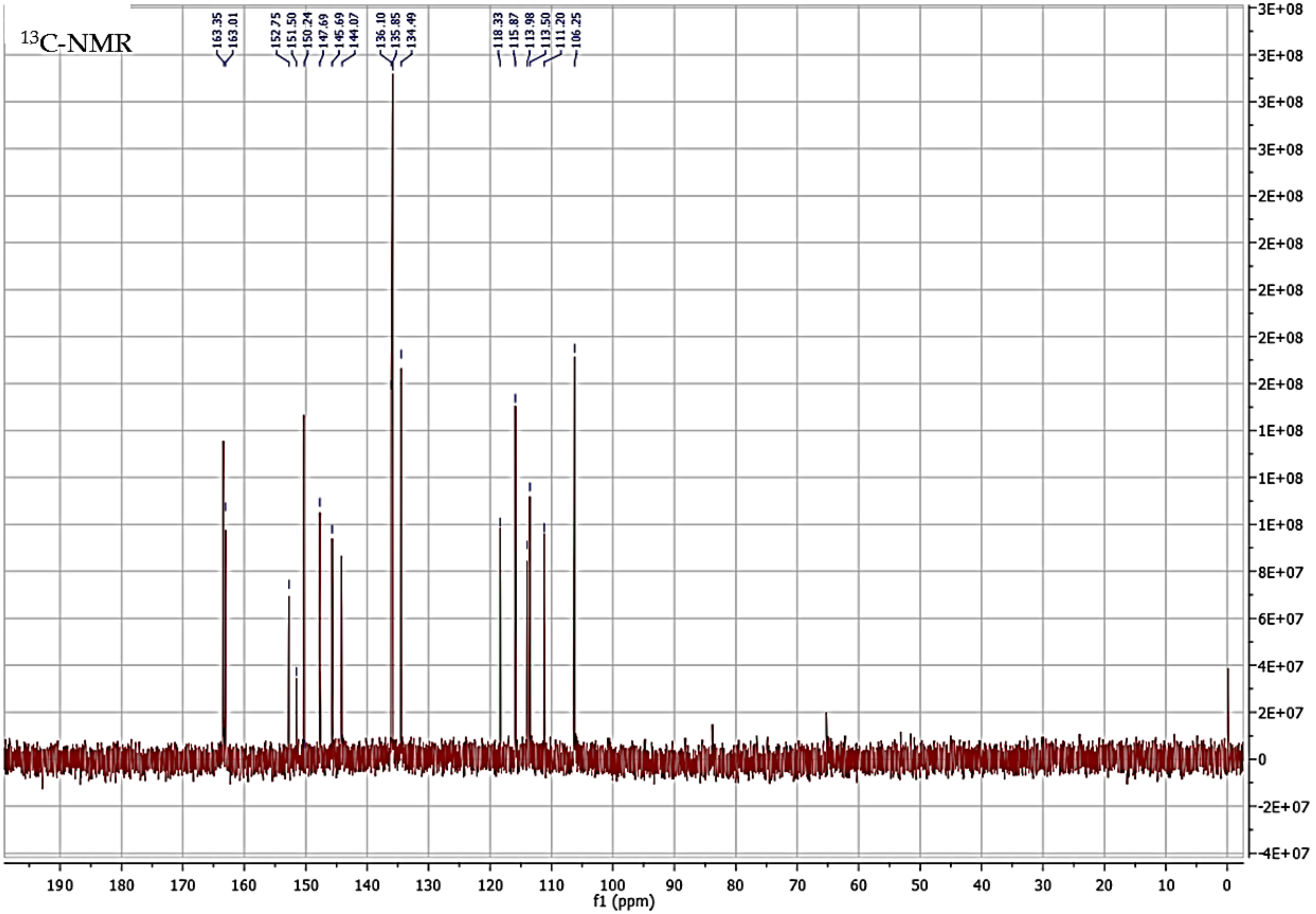

^{13}C-NMR
163.35
163.01
152.75
151.50
150.24
147.69
145.69
144.07
136.10
135.85
134.49
118.33
115.87
113.98
113.50
111.20
106.25
f1 (ppm)

E)

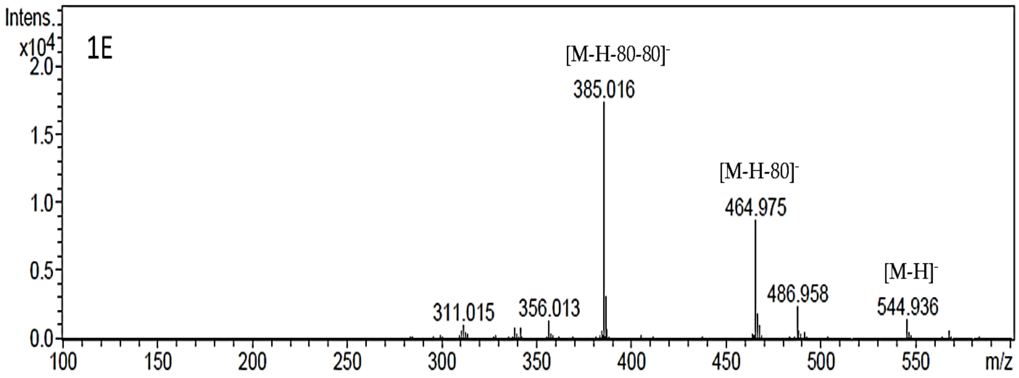

Figure S3: A-D) NMR spectra and 2 E) HR-ESI–MS spectra for Compound **2**:

7-dihydroxycoumarin-3,6-disulfate

A)

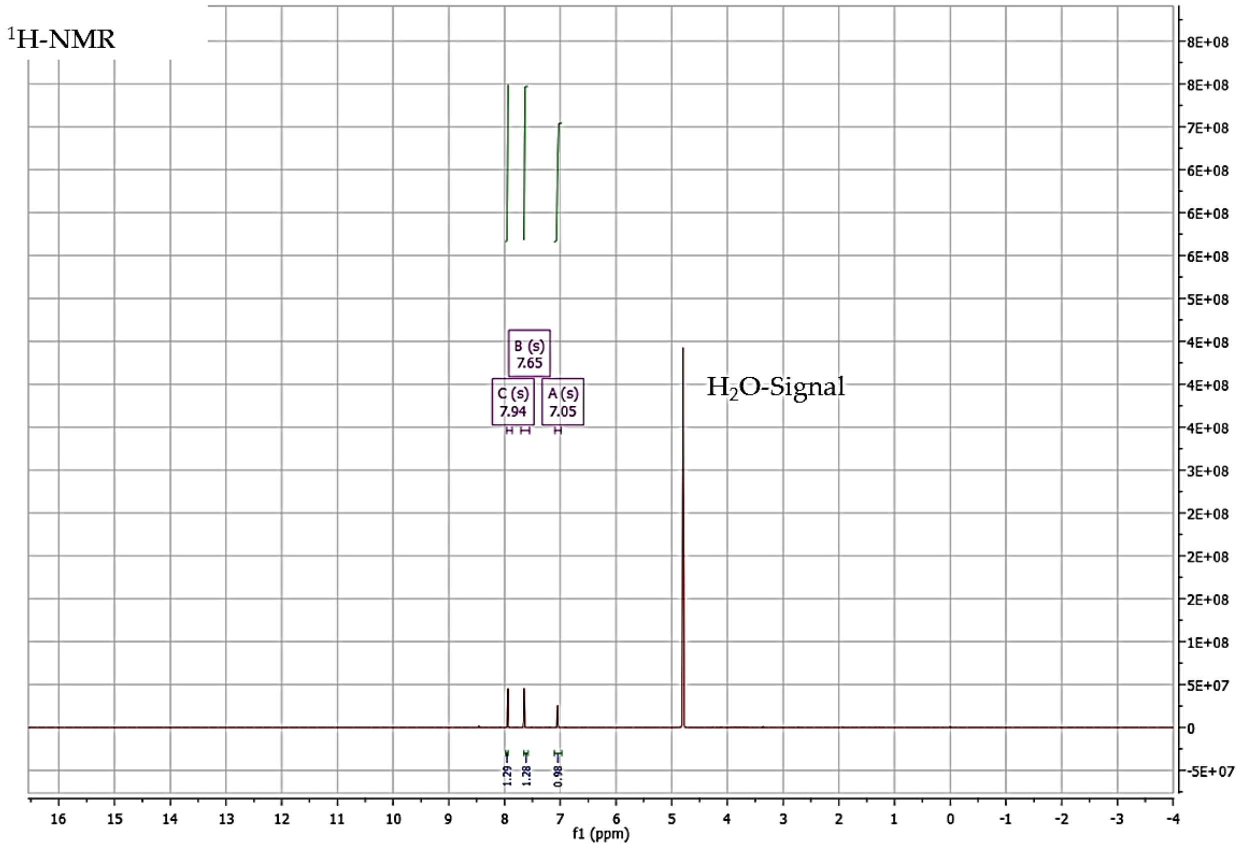

B)

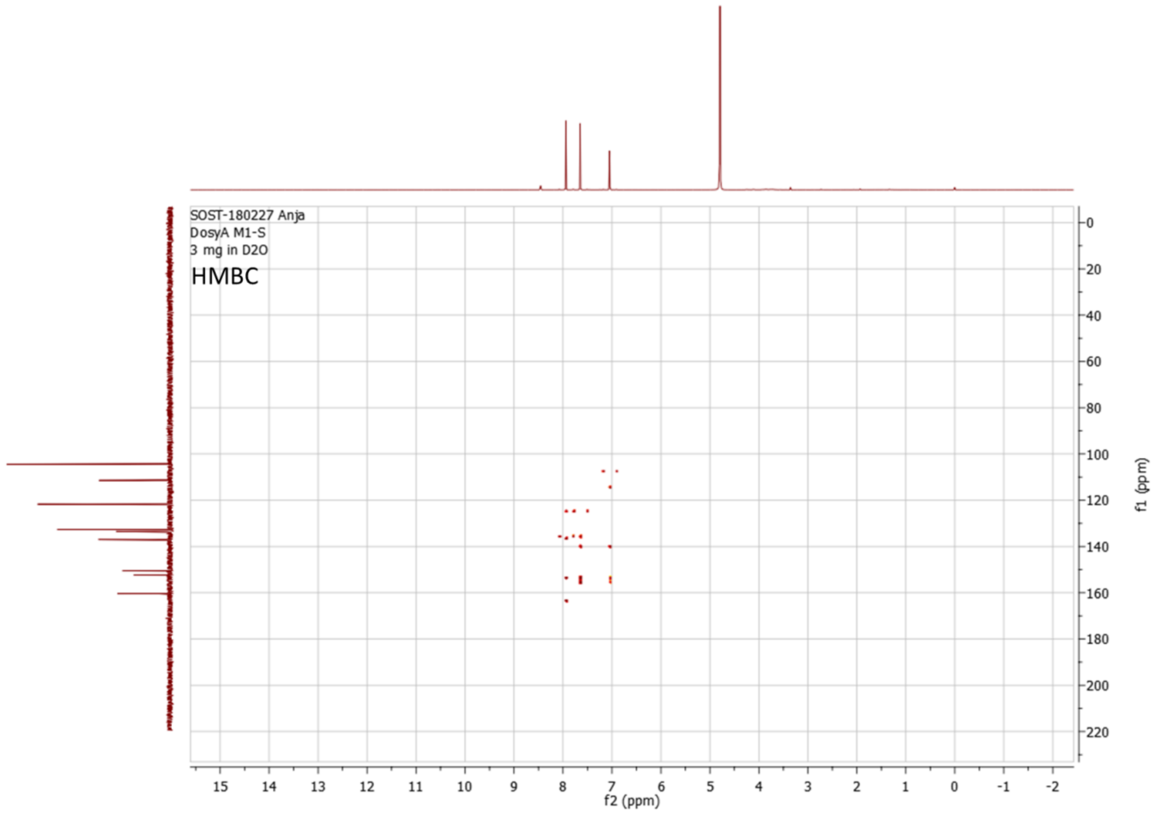
SOST-180227 Anja
DosyA M1-S
3 mg in D2O
HMBC
f1 (ppm)
f2 (ppm)

C)

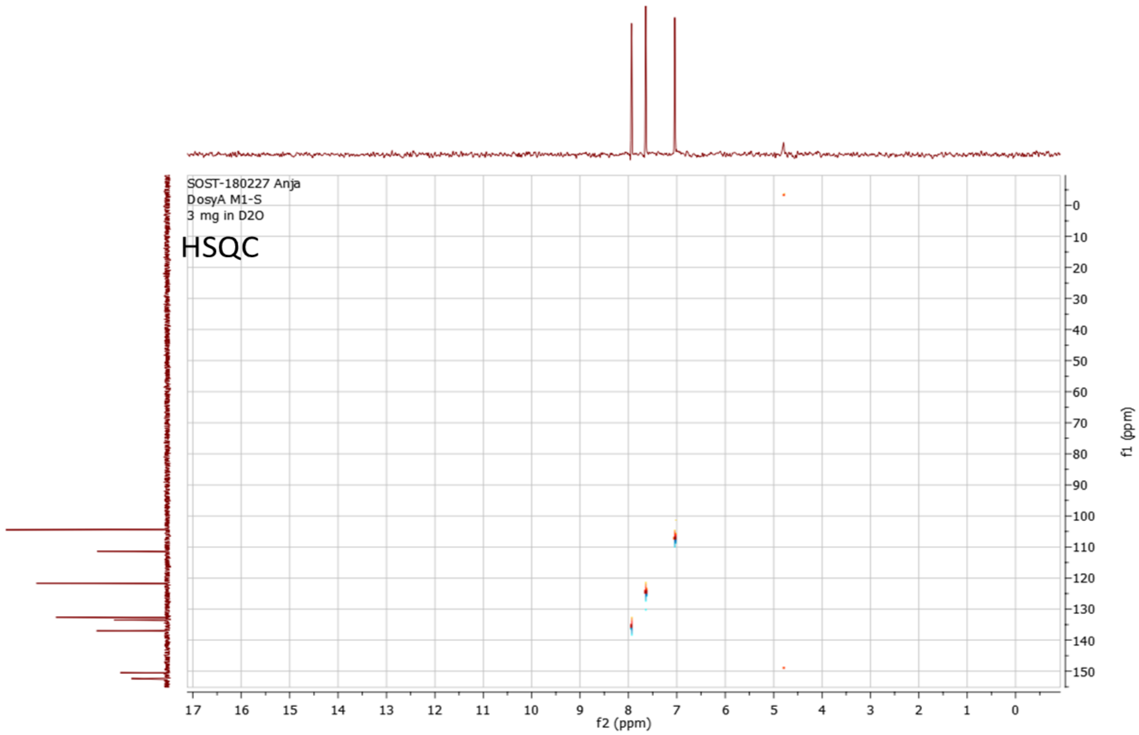
SOST-180227 Anja
DosyA M1-S
3 mg in D2O
HSQC
f1 (ppm)
f2 (ppm)

D)

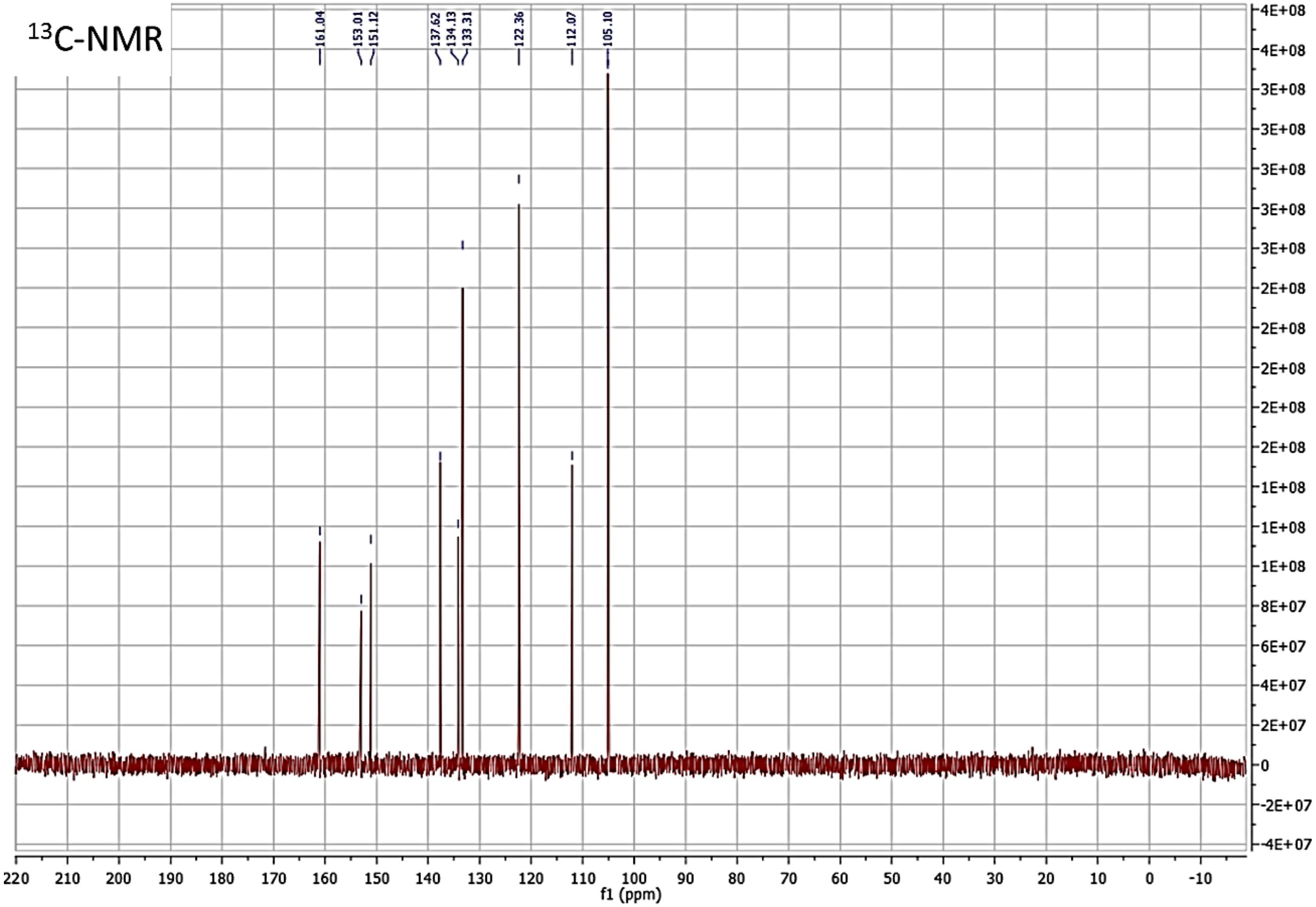

^{13}C-NMR
161.04
153.01
151.12
137.62
134.13
133.31
122.36
112.07
105.10
f1 (ppm)

E)

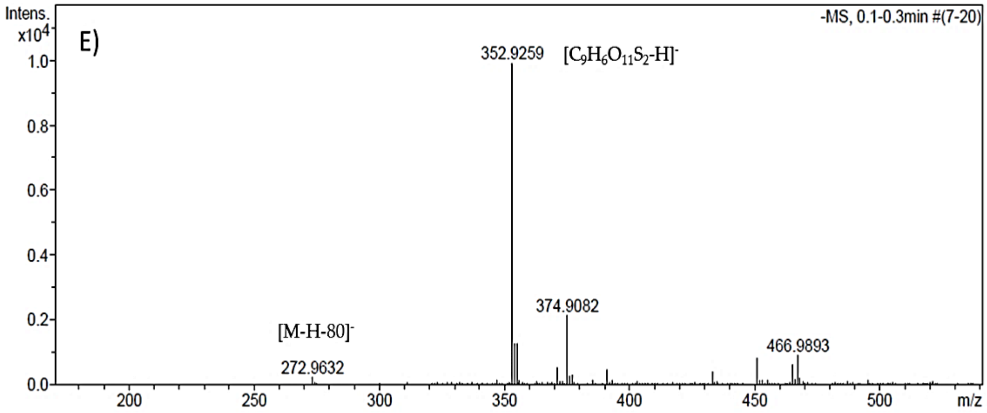

Intens.
x10^4
E)
-MS, 0.1-0.3min #(7-20)
352.9259
[C9H6O11S2-H]-
374.9082
[M-H-80]-
272.9632
466.9893
1.0
0.8
0.6
0.4
0.2
0.0
200
250
300
350
400
450
500
m/z

Figure S4: UV spectra of the 4 Coumarins recorded on line by DAD

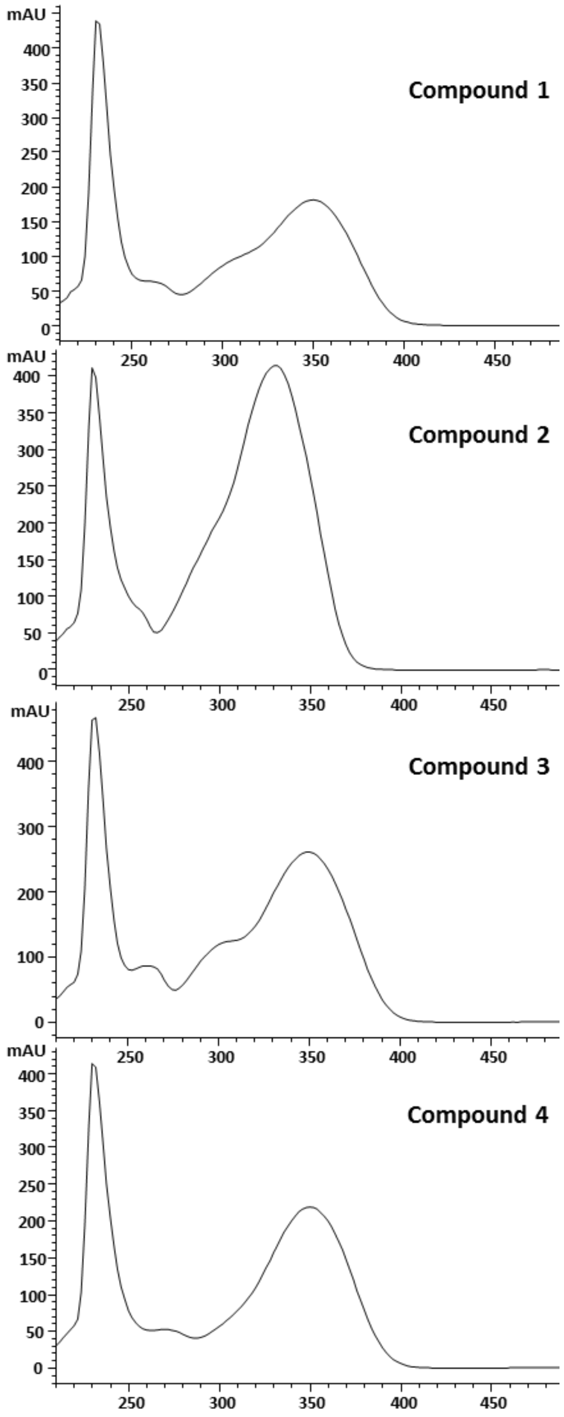

Figure S5: Determination of coumarins (**1**: dasycladin A, **2**: dasycladin B, **3**: 6,7-dihydroxycoumarin-3sulfate, **4**: 3,6,7-trihydroxycoumarin) in the marine green alga *Dasycladus vermicularis*; chromatograms were recorded at 254 nm and 350 nm sample DV-1, the other traces show the assignment of individual compounds by LC-MS in EIC mode.

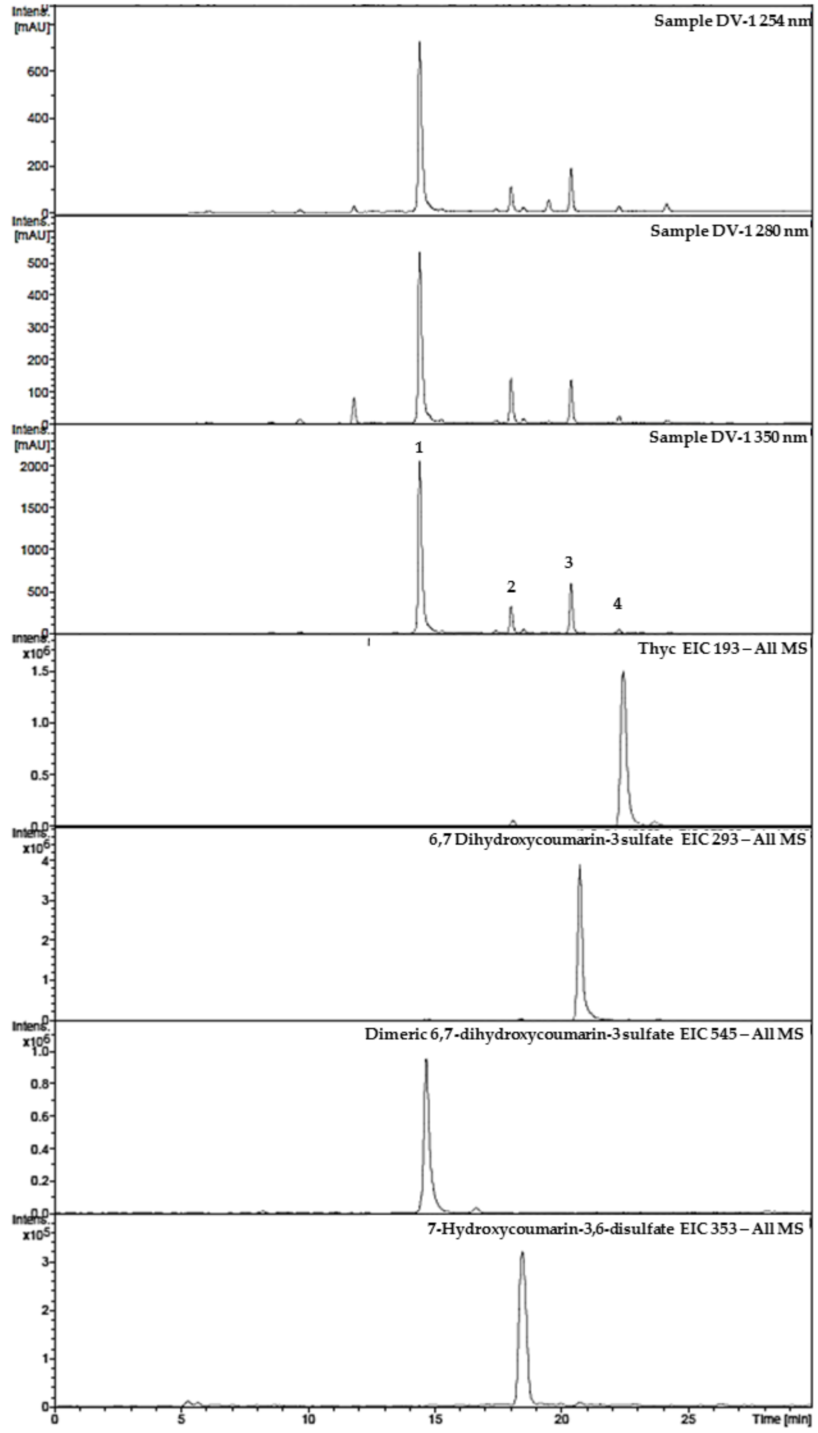

27 The Access and Benefit-Sharing Clearing-House. Available online: https://absch.cbd.int/countries/GR (accessed on 13 July 2018).
28 Convention on Biological Diversity. Available online: https://www.cbd.int/ (accessed on 13 July 2018).
29 Pérez-Rodríguez, E.; Gómez, I.; Karsten, U.; Figueroa, F.L. Effects of UV radiation on photosynthesis and excretion of UV-absorbing pigments of *Dasycladus vermicularis* (Chlorophyta, Dasycladales) from Southern Spain. *Phycologia* **2008**, *37*, 379–387.
